# Long-Term Success With a TDM-1 Biosimilar in Recurrent HER2-Positive Breast Cancer With Multisite Metastases: A Comprehensive Case Study

**DOI:** 10.7759/cureus.67047

**Published:** 2024-08-16

**Authors:** Ghanashyam Biswas, Ganesh C Subudhi, Sutapa Biswas, Bharat Das

**Affiliations:** 1 Oncology, Sparsh Hospital, Bhubaneswar, IND; 2 Pathology, Sparsh Hospitals and Critical Care, Bhubaneswar, IND; 3 Palliative Care, Sparsh Hospitals and Critical Care, Bhubaneswar, IND

**Keywords:** trastuzumab emtansine (t-dm1), brain metastasis, breast cancer, recurrence, biosimilar

## Abstract

This case report highlights the treatment approaches for breast cancer with metastasis to multiple sites, including the brain, liver, lungs, and bones. It also explores the role of trastuzumab emtansine (T-DM1) and its biosimilar as targeted therapies for HER2-positive breast cancer in long-term treatment. A 54-year-old postmenopausal female patient with recurrent breast cancer and metastasis was treated with trastuzumab and paclitaxel (12 cycles), followed by trastuzumab maintenance therapy (71 cycles). She received radiation therapy of 30 Gy in 10 fractions after presenting with bleeding from the breast lesion. Oral therapy with lapatinib and letrozole was prescribed for a year to treat the breast cancer, along with the lesions and enhanced nodules observed in the patient’s lungs, liver, and bones. A year later, she presented with complaints of seizures and loss of consciousness and was diagnosed with brain metastasis. Whole-brain radiation therapy (WBRT) was administered after performing contrast-enhanced computed tomography (CECT). The WBRT was followed by trastuzumab emtansine (T-DM1) biosimilar therapy. Currently, the patient has received more than 30 cycles of T-DM1 and has survived while maintaining a good quality of life. The patient has survived 7.5 years in a stable disease condition after the detection of recurrent breast cancer with metastasis to the liver, lungs, bones, and brain. This case report demonstrates the long-term efficacy of T-DM1 and its satisfactory safety profile. Overall, it provides valuable insights into the management of and challenges faced during the treatment of recurrent breast cancer with metastasis.

## Introduction

Breast cancer is the most prevalent cancer in women, resulting in 670,000 deaths worldwide in 2022 alone, according to the World Health Organization [1]. Understanding the type and stage of breast cancer, as well as the severity of metastasis, is crucial for strategizing treatment options. Clinicians adopt multiple therapy approaches to prevent the recurrence of cancer, including surgical removal of the tumor to eliminate the primary source, radiation therapy to lower the risk of recurrence in surrounding tissues, and hormonal or chemotherapy to inhibit the proliferation of cancer cells [1]. The advancement in targeted therapy has shown more effective and less toxic management of specific types of breast cancer, offering new hope to patients suffering from the disease. Trastuzumab emtansine (T-DM1), an antibody-drug conjugate (ADC), combines the efficacy of trastuzumab, a monoclonal antibody that targets HER2-positive breast cancer, with a cytotoxic microtubule inhibitor, DM1, which helps deliver the drug precisely to the cancer cells through receptor-mediated endocytosis, making it a potential treatment for HER2 (now erbB2)-positive patients [2,3].

To improve the accessibility of this ADC in terms of cost and availability, a biosimilar of T-DM1 was developed. Its efficacy, safety, and immunogenicity were approved by the Drug Controller General of India (DCGI) based on a phase 3 clinical trial, making it the world’s first approved biosimilar of T-DM1 [4]. Herein, we present a case of a breast cancer patient with recurrent disease and metastases in the lungs, liver, bones, and brain, who achieved long-term survival after treatment with radiotherapy and a T-DM1 biosimilar.

## Case presentation

A 54-year-old woman was diagnosed with breast cancer in November 2015, presenting with a heterogeneously enhancing mass measuring 3.3 x 2.1 cm in her left breast, along with thickening of the associated skin and metastatic axillary nodes (the largest measuring 3.6 x 2.4 cm) observed on chest computed tomography (CT). She had metastatic disease with enhanced lesions in both lobes of the liver (3.6 x 3.3 cm) and enlarged nodules in both upper lobes of the lung parenchyma and right hilum. The Tc-99m methyl diphosphate (MDP) bone scan of the skeletal system showed focal low-grade tracer uptake in the left proximal femur, indicating bone metastasis. Histopathological examination revealed infiltrating ductal carcinoma (Grade III) in the breast core biopsy, a positive estrogen receptor of 80%, a negative progesterone receptor, and a positive c-erbB2 score of 3+ in her immunohistochemistry profile.

The treatment commenced with chemotherapy sessions (12 cycles) that included a weekly regimen of paclitaxel (150 mg in 500 mL NS) and trastuzumab (160 mg in 250 mL NS) from November 2015 to January 2016. The complete blood count (CBC), renal function test (RFT), and liver function test (LFT) were repeated before every alternate cycle. A WBC count >3500/mm³, ANC >1500/cm, HB >8 g%, and platelet count >100,000/mm³ were considered optimal before starting the next cycle. The patient was then placed on trastuzumab maintenance therapy at a dose of 440 mg for 71 cycles from February 2016 to February 2020. A CT scan after maintenance therapy showed grossly stable skin thickening with a mild increase in small nodules. No enlarged axillary nodes were detected in the breast, and the previously detected enlarged nodules in the lungs and hilum were stable. There was no significant change in liver lesions. Reports of a 2D echocardiogram showed maintained left ventricle ejection fraction (LVEF) at 78%.

**Figure 1:**
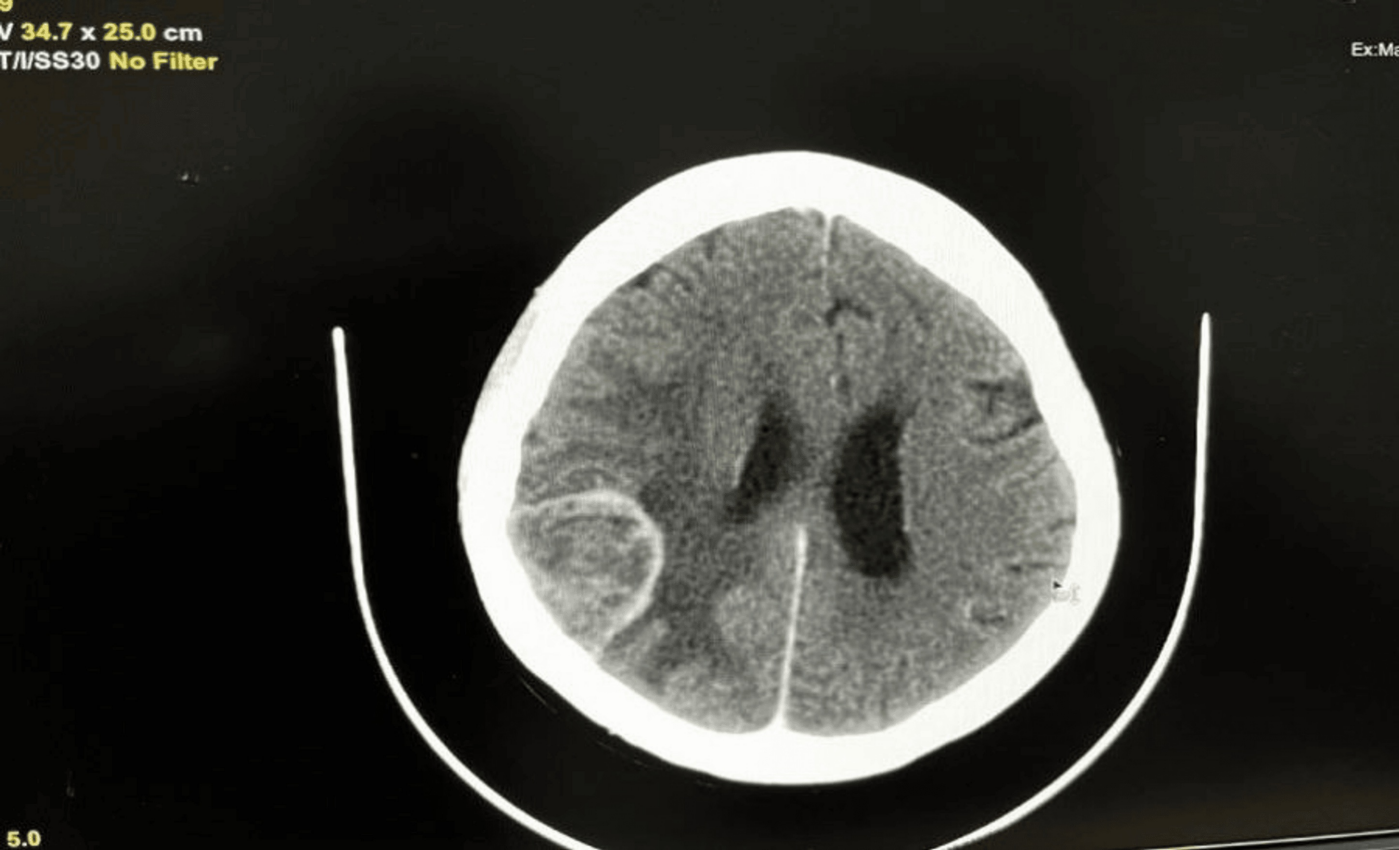
A heterogeneous enhancing solid mass lesion of size 41 x 30 mm in the right posterior parietal region with moderate surrounding edema suggestive of metastasis as observed via CECT CECT, contrast-enhanced computed tomography.

A few days later, the patient presented with bleeding over the right central lesion. She was referred to a radiation oncologist for adjuvant therapy and supportive care, where palliative radiotherapy was advised in March 2020 at a dose of 30 Gy in 10 fractions, along with a boost of 9 Gy in 5 fractions. Additionally, oral therapy with lapatinib at 500 mg BD and letrozole at 2.5 mg OD was recommended, along with three-monthly injections of zoledronic acid. A year after beginning oral therapy, the patient presented with headaches, loss of consciousness, vomiting, and seizures. A brain contrast-enhanced computed tomography (CECT) in February 2021 revealed the presence of a heterogeneously enhancing solid mass lesion in the right posterior region with surrounding perilesional edema, indicating brain metastasis (Figure 1). Whole-brain radiation therapy (WBRT) was administered to treat the brain metastasis, followed by T-DM1 therapy and letrozole.

**Figure 2:**
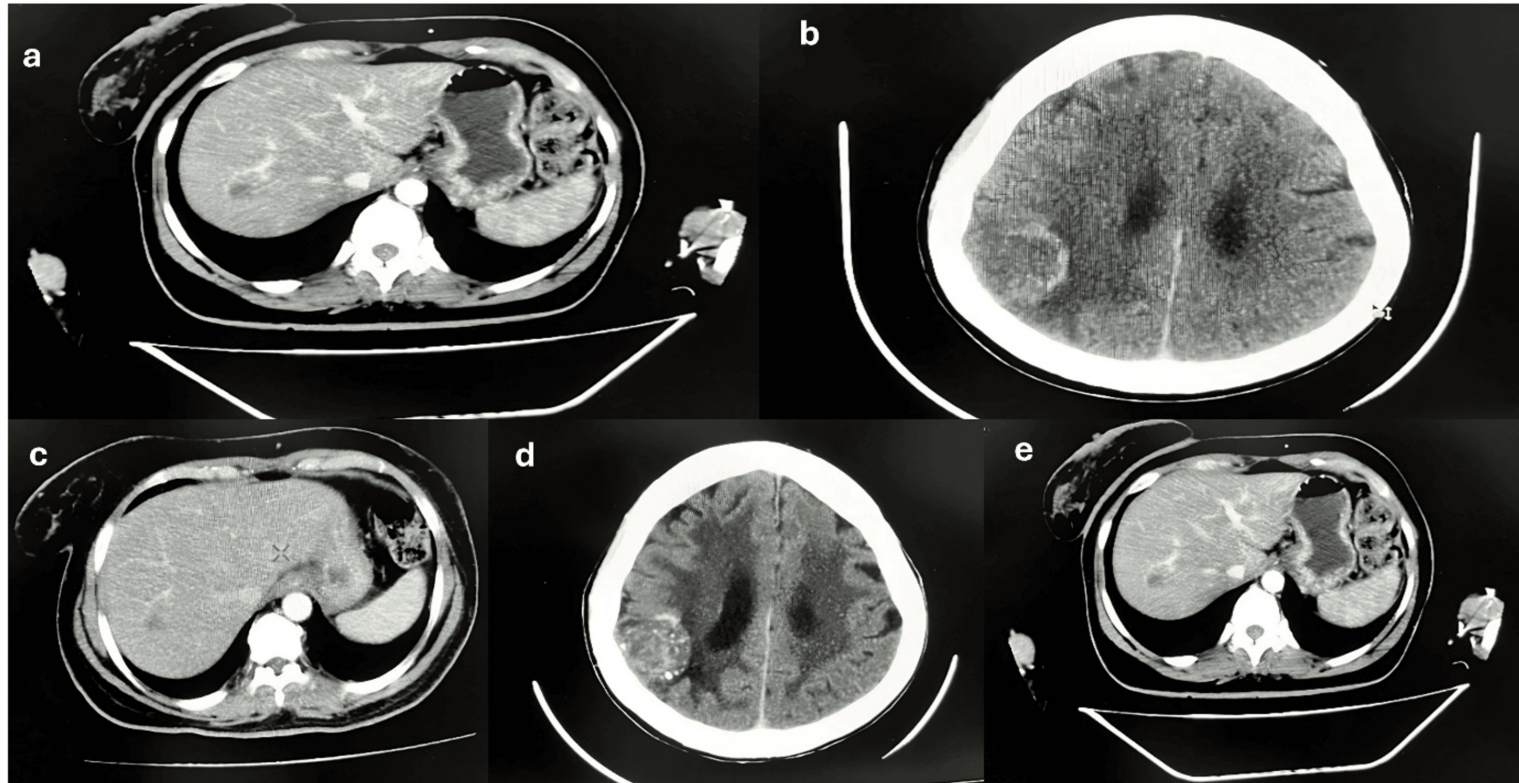
(a) A small enhancing nodule measuring 13 mm in the right lobe in segment VII identified on CECT; (b) a heterogeneously enhancing solid mass lesion measuring 34 x 32 mm in the right post-parietal region with perilesional edema suggestive of a regressed lesion identified via CECT; (c) stable disease observed on CECT; (d and e) both images suggestive of stable disease as observed on CECT. CECT, contrast-enhanced computed tomography.

The patient received the first cycle of T-DM1 (245 mg, Q3W) with the innovator brand in May 2021. Later, with the immediate launch in India, a biosimilar of T-DM1 (Ujvira^TM^, Cadila Healthcare Limited, Ahmedabad, India) was recommended, considering financial and logistical concerns. T-DM1 therapy showed a partial response, with regressed lung and breast nodules and no new lesions; however, liver and bone metastasis persisted, along with vertebral body degenerative changes (Figure 2).

**Figure 3:**
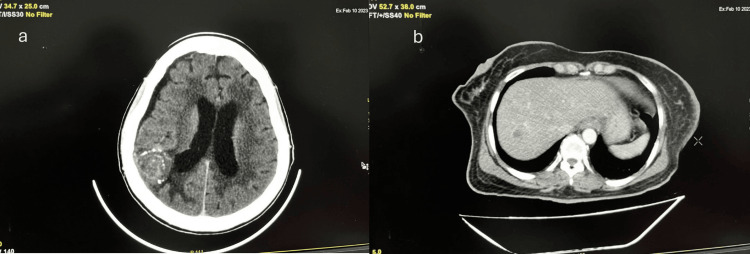
(a) A 35 x 28 mm lesion in the right post-parietal lobe with perilesional edema suggestive of stable disease via CECT; (b) A 12 x 12 mm small non-enhancing hypodense lesion in the right lobe in segment VII suggestive of a stable lesion observed on CECT. CECT, contrast-enhanced computed tomography

The reports of 2D echocardiography showed maintained LVEF results at 54%. Currently, the patient has received more than 30 cycles of third-line therapy with T-DM1 along with paclitaxel and letrozole and has survived 7.5 years in a stable disease condition after the detection of recurrent breast cancer with liver, lung, bone, and brain metastasis, while maintaining a good quality of life to date (Figure 3).

## Discussion

This report describes a case of a postmenopausal female patient with metastatic recurrent breast cancer. Chemotherapy with paclitaxel and trastuzumab for three months was the first line of treatment offered, followed by trastuzumab maintenance therapy for one month. This treatment approach exhibited a mild increase in the small nodules of the breast with no significant difference in the metastasis of the lungs, liver, and bone. These results align with the previously conducted clinical trial by Furukawa et al., where combination therapy of paclitaxel and trastuzumab had a synergistic effect and a better response rate compared to either paclitaxel or trastuzumab alone [5]. Furthermore, the patient was kept on oral therapy of lapatinib and letrozole for almost a year. The phosphorylation-inhibiting property of lapatinib allows it to retain antitumor activity in cancerous cells that have become resistant to trastuzumab. This combination therapy has shown relatively greater benefit and prolonged progression-free survival in HER2-positive patients with metastasis in the liver or more than three sites, especially in postmenopausal women. The results of repeated CBC, 2D echocardiography, electrocardiogram, ultrasonography, and X-ray [6,7] before each follow-up were in line with the aforementioned research, demonstrating that the combination oral therapy of lapatinib and letrozole was well tolerated by the patient with an insignificant decrease in quality of life for almost a year.

An important issue in patients suffering from breast cancer is the incidence of central nervous system metastasis, especially brain metastasis, which results in poor morbidity, reduced survival, and impaired quality of life [8]. Conventionally, surgery and radiation therapy were the available treatment options, but recent guidelines from the American Society of Clinical Oncology (2018) [9] and the European Society for Medical Oncology (2021) [10] have recommended the use of targeted therapy along with radiation for the treatment of breast cancer patients with brain metastasis for better response. In the presented case, an enhanced solid mass in the brain was observed in the brain CECT, which was treated with WBRT followed by targeted therapy with T-DM1. The innovator brand was used during the initial cycles and then replaced with the biosimilar drug to make it more affordable for the patient.

A preclinical study conducted by Adams et al. revealed that T-DM1, being a radiosensitizer, can prolong tumor control when administered with radiotherapy in HER2-positive cancer patients [11]. This was further confirmed by preliminary data from breast cancer patients with brain metastasis reported by Géraud et al. The data showed no increased toxicity and good control of symptoms even after three months of treatment [12]. Although T-DM1 has nearly halved the risk of recurrence and demonstrated improved progression-free survival and increased survival rates, combination therapy with radiation therapy has raised concerns regarding toxicity. Similar concerns were raised in a meta-analysis conducted by Salvestrini et al., which provided an overview of studies using a combination of T-DM1 and RT (radiation therapy) for treating early and advanced breast cancer. The data indicated that the safety profile of this combination was suitable for treating nonmetastatic breast cancer compared to metastatic cancer. Treatment-related adverse events include radionecrosis, neurological symptoms, emesis, fatigue, and alopecia when the RT site is the brain [13]. Our study did not observe these symptoms because concurrent RT with T-DM1 was not the treatment approach. The therapy with RT followed by multiple cycles of T-DM1 showed regression in the breast cancer nodule with no new lesions in the brain. These results align with the subgroup analysis of the EMILIA trial conducted on breast cancer patients with brain metastasis [14,15].

Conclusively, the long-term use of T-DM1 therapy exhibited a satisfactory profile, with one significant exception: a brief period of poor R wave progression shown in the ECG after approximately nine cycles of T-DM1, which may indicate the existence of left ventricular (LV) dysfunction or diabetic cardiomyopathy. LV dysfunction has been reported as a cardiotoxic consequence of T-DM1 in the LHORA study, particularly in breast cancer patients taking trastuzumab as the first-line treatment [16,17]. Poor R wave progression can also be attributed to diabetic cardiomyopathy [18], as the patient presented with hyperglycemia and was treated with insulin infusion.

## Conclusions

We report a case of recurrent breast cancer in a postmenopausal woman with liver, lung, bone, and brain metastasis. The patient was able to achieve long-term survival with an insignificant decrement in quality of life following sequential treatment with trastuzumab, lapatinib, letrozole, radiotherapy, and T-DM1 over a period of more than seven years. Future research should focus on optimizing treatment sequences and exploring new therapeutic combinations to enhance efficacy and minimize side effects. Physicians can draw insights from this case to guide treatment plans and maintain quality of life, thereby advancing the standard of care for metastatic breast cancer.
